# Supplementary material for: Hollow Protein Fibers Templated Synthesis of Pt/Pd Nanostructures with Peroxidase-like Activity
Source: Viruses. 2025 Dec 16;17(12):1627. doi: 10.3390/v17121627 (PMC12737335; doi:10.3390/v17121627)
Supplement: Supplementary file 1 [file viruses-17-01627-s001.zip › viruses-3956502-supplementary.pdf]

Hollow Protein Fibers Templated Synthesis of Pt/Pd Nanostructures with Peroxidase-like Activity

Beizhe Huang, Mengting Fan, Yuhan Li, Ting Zhang and Jianting Zhang

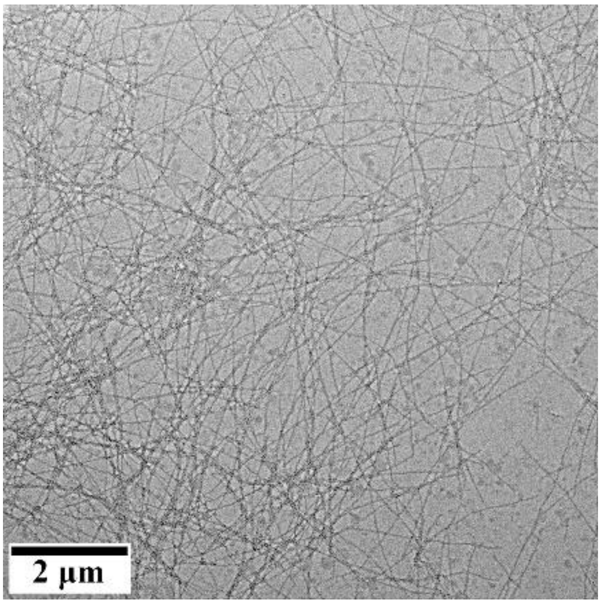

Figure S1. TEM image of TMVFs after incubated at 70 °C for 1 h.

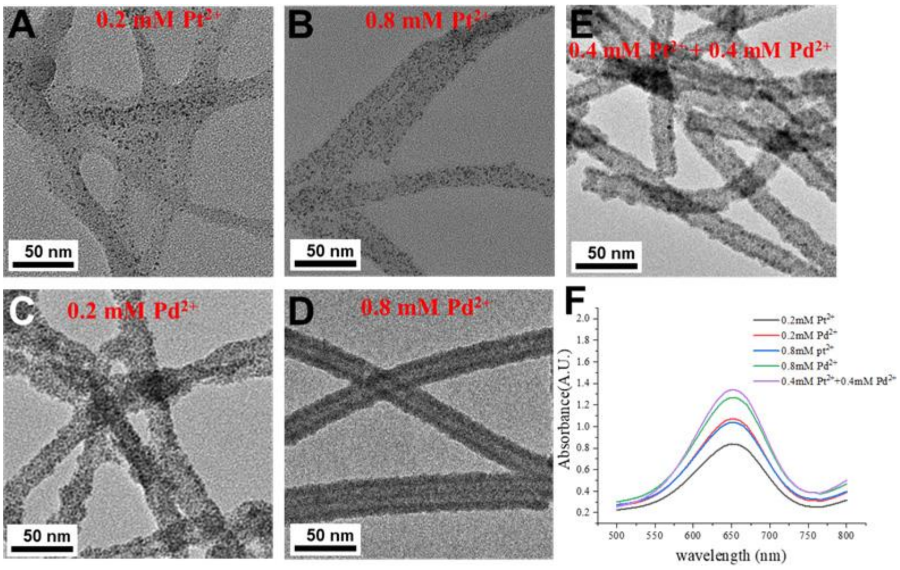

Figure S2. Characterization of Pt or Pd deposition on the TMVF with different concentration. (A-E) TEM images of the Pt, Pd or bimetallic NW prepared with different concentration of metal ions. (F) UV-vis spectra of TMB system catalyzed by Pt, Pd or bimetallic NW prepared with different concentrations of metal ions.

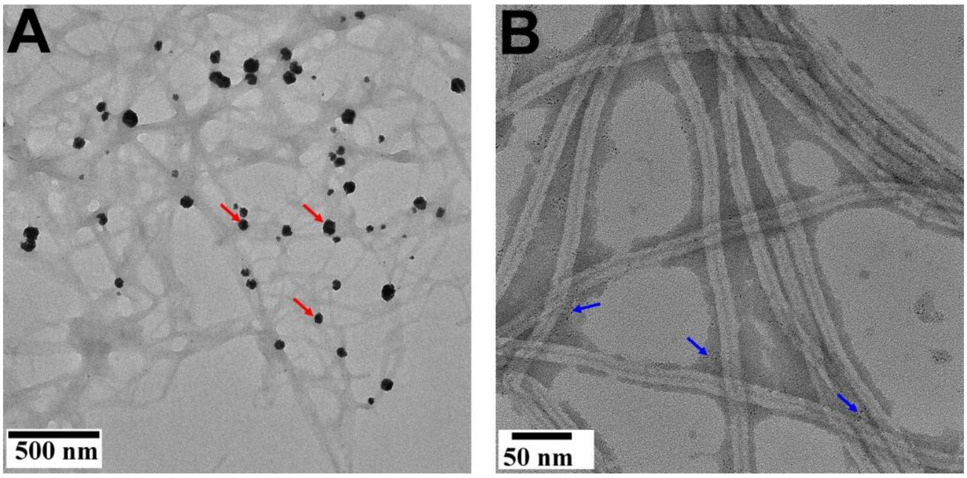

Figure S3. TEM images showed random nucleation of Ag^{+} (A) and Pt^{2+} (B) unlike Pd^{2+} which site-specially nucleated in the channel of TMVF.

Table S1. Comparison of kinetic parameters of different Pt- or Pd-based nano-enzymes

Catalyst	Substrate	Km (mM)	Vmax ($\times 10^{-7}$, M/s)
TMV/PtNPs[1]	H_2O_2	140	0.519
	TMB	0.15	0.243
Pd@Pt core-frame nanodendrities[2]	H_2O_2	14	0.9
	TMB	0.43	0.51
Pd@ZIF-8[3]	H_2O_2	14.02	2.77
	TMB	0.13	3.07
Pd nanostructures[4]	H_2O_2	1064	44.3
	TMB	0.16	20.1
This work	H_2O_2	9.57	0.313
	TMB	0.11	2.102

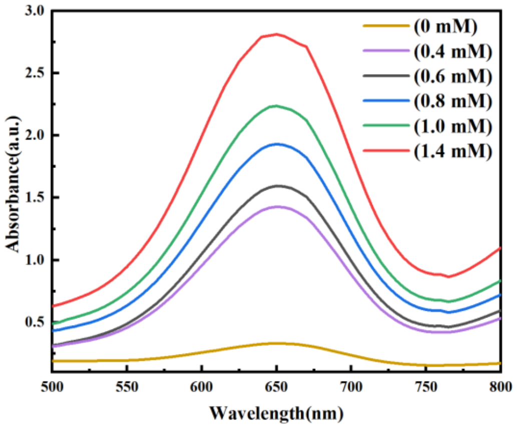

Figure S4. UV-vis spectra of TMB system catalyzed by Pt/Pd prepared with different concentrations of metal ions (Pd^{2+}: Pt^{2+}=1:1).

References

1. Guo J., Zhao X., Hu J., Lin Y., Wang Q. Tobacco Mosaic Virus with Peroxidase-Like Activity for Cancer Cell Detection through Colorimetric Assay. Mol. Pharmaceutics **2018**, 15, 2946-2953.
2. Li Y., Li S., Bao M., Zhang L., Carraro C., Maboudian R., Liu A., Wei W., Zhang Y., Liu S. Pd Nanoclusters Confined in ZIF-8 Matrixes for Fluorescent Detection of Glucose and Cholesterol. ACS Appl. Nano Mater. **2021**, 4, 9132-9142.
3. Ge C., Wu R., Chong Y., Fang G., Jiang X., Pan Y., Chen C., Yin J.-J. Synthesis of Pt Hollow Nanodendrites with Enhanced Peroxidase-Like Activity against Bacterial Infections: Implication for Wound Healing. Adv. Funct. Mater. **2018**, 28, 1801484.
4. Gao Z., Hou L., Xu M., Tang D. Enhanced Colorimetric Immunoassay Accompanying with Enzyme Cascade Amplification Strategy for Ultrasensitive Detection of Low-Abundance Protein. Scientific Reports **2014**, 4, 3966.
